# Dietary Polyphenols as Natural Modulators of NF‐κB Signaling in Inflammation‐Driven Non‐Communicable Diseases: Focus on Cancer

**DOI:** 10.1002/fsn3.72027

**Published:** 2026-07-02

**Authors:** Khuzin Dinislam, Anas Shamsi, Syed Tasqeruddin, Moyad Shahwan

**Affiliations:** ^1^ Department of General Chemistry Bashkir State Medical University Ufa Russia; ^2^ Center of Medical and Bio‐Allied Health Sciences Research Ajman University Ajman UAE; ^3^ Department of Pharmaceutical Chemistry, College of Pharmacy King Khalid University Abha Saudi Arabia; ^4^ Clsenter of Excellence in Precision Medicine and Digital Health Chulalongkorn University Bangok Thailand; ^5^ Department of Clinical Sciences College of Pharmacy and Health Sciences, Ajman University UAE

**Keywords:** cancer, dietary polyphenols, inflammation, oncogenic signaling, oxidative stress, phytochemicals

## Abstract

Chronic low‐grade inflammation is a key driver of non‐communicable diseases (NCDs), including cancer, cardiovascular disorders, metabolic syndrome, and neurodegenerative diseases (NDs). Nuclear factor kappa‐light‐chain‐enhancer of activated B cells (NF‐κB) is a master transcription factor that orchestrates inflammatory signaling, immune dysregulation, cellular proliferation, and survival pathways implicated in inflammation‐driven pathologies. Initiation and activation of other chronic inflammatory diseases can be linked to continued NF‐κB activation, thus making it an attractive target for therapeutics. Dietary polyphenols that are found in fruits, vegetables, tea, coffee, and other foods have a broad therapeutic potential and have emerged as potent natural modulators of NF‐κB signaling. Various studies have highlighted the relevance of different classes of polyphenols. These classes, flavonoids, phenolic acids, and other polyphenolic compounds exert anti‐inflammatory and chemopreventive effects through multiple mechanisms, by suppressing NF‐κB activation, inhibition of kinases, reduction of oxidative stress, and modulation of epigenetic and redox‐sensitive pathways. The present review provides insight into the mechanisms by which dietary polyphenols regulate NF‐κB signaling and highlights their role in the prevention of inflammation‐driven NCDs.

## Graphical abstract

Dietary polyphenols and phytochemicals suppress NF‐κB signaling. Mechanistically, by inhibiting IκBα degradation and preventing nuclear translocation of p65/p50 complexes, the polyphenols reduce the expression of pro‐inflammatory and pro‐survival genes, thereby decreasing overall cancer cell survival.

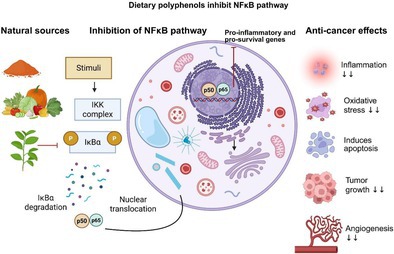

## 1 Introduction

Nuclear factor kappa B (NF‐κB) signaling pathway is one of the most extensively studied and biologically important signaling pathways. well‐studied and crucial pathways. David Baltimore and his colleagues discovered it in 1986 (Sen and Baltimore 1986). Initially, it was identified and recognized as one of the transcription factors that bind to the enhancer region of the immunoglobulin kappa light chain in B cells (Sen and Baltimore 1986). NF‐κB is highly conserved, having key roles associated with various cellular responses in humans, such as stress, cytokines, and pathogens. Due to such vast roles, this is a key focus in cancer and other inflammatory diseases (Hayden and Ghosh 2012). It consists of two monomers that remain linked together. Its function is strictly guided by various inhibitory proteins, mainly belonging to Inhibitors of κB (IκBs)Phy, which sequester it in the cytoplasm until the activation signals trigger its nuclear translocation (Karin and Ben‐Neriah 2000). As it is involved in diverse biological processes, it is essential for maintaining body function at the cellular level.

**FIGURE 1:**
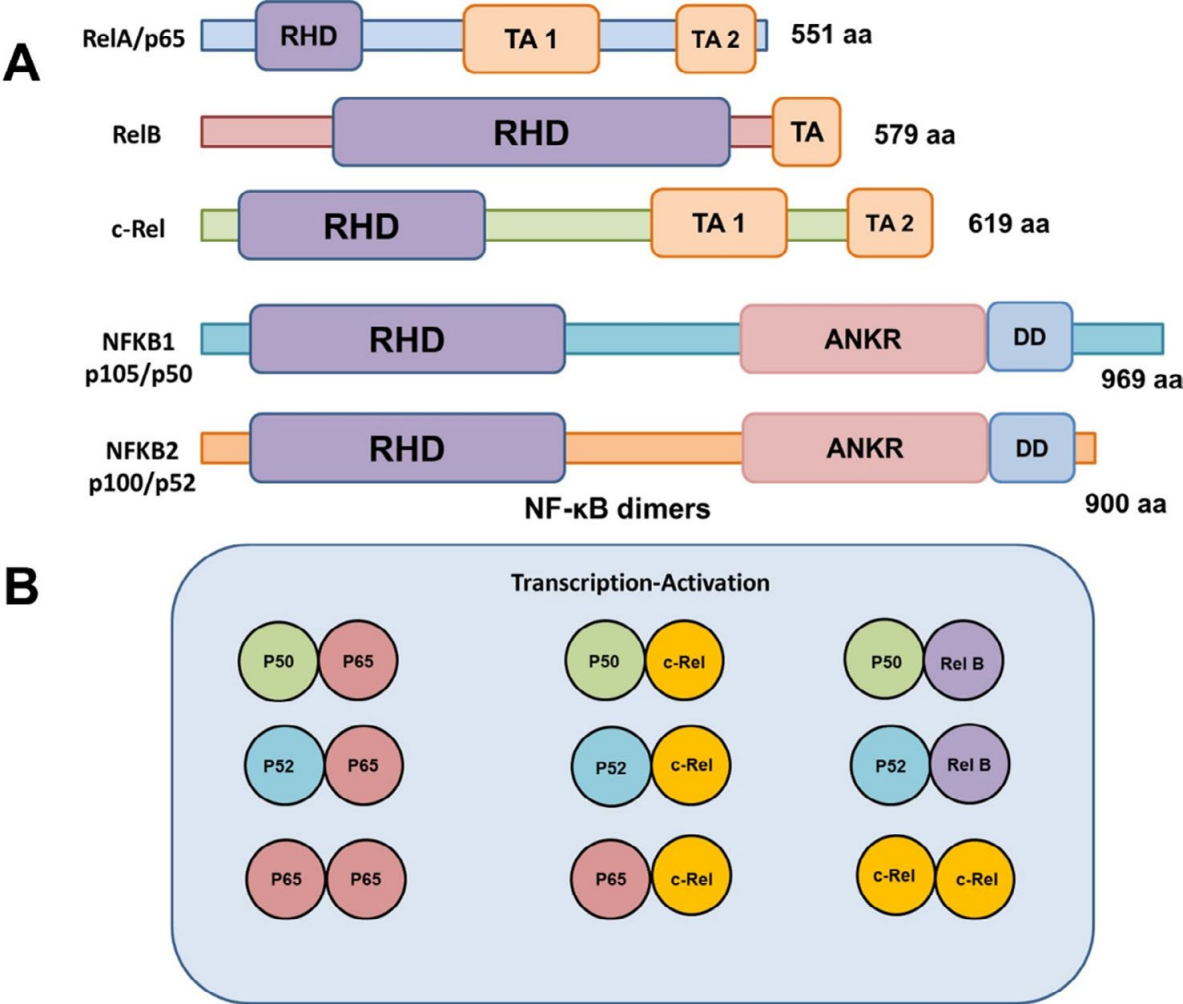
Structural organization and transcriptional activation of NF‐κB family members: (A) Domains and structure of NFκB subunits; RHD: RelA homology domain; ANKR: Ankyrin repeat domain; TA: Transactivation Domain. (B) NF‐κB Dimer Combinations for transcriptional activation. This panel illustrates various dimeric combinations of NF‐κB subunits (p50, p52, RelA/p65, RelB, and c‐Rel) involved in transcriptional activation. Functional dimers form through the pairing of subunits with or without intrinsic transactivation domains, regulating gene expression in response to diverse cellular stimuli.

NF‐κB is constitutively expressed in most cell types but remains inactive in the cytoplasm under basal conditions dormant state has NF‐κB, which is conserved across all species. It only moves to the nucleus when activated, where it controls the production of several genes related to inflammation, growth, and immunity. NF‐κB is persistently active in several pathological conditions, including cancer, arthritis, chronic inflammation, neurodegeneration, and cardiovascular diseases. Five distinct NF‐κB family members have been identified: NF‐κB 1 (p50/p105), NF‐κB2 (p52/p100), RelA (p65), RelB, and c‐Rel, as shown in Figure 1A,B. The canonical and non‐canonical NF‐κB activation routes have been distinguished. Before the translocation of the NF‐κB complex into the nucleus, NF‐κB 1 and NF‐κB 2 proteolytically processed into the active p50 and p52 subunits, respectively (Aggarwal 2003). NF‐κB, which is made up of p50 and RelA, is inactive in the cytoplasm when cells are at rest. It is linked to several inhibitory molecules, such as IkB family, p105, and p100, of which IkBα is the most prevalent. Phosphorylation of two conserved serine (S) residues in the N‐terminal domain of IkB proteins activates the inactive NF‐κB/IkB complex (Strickland and Ghosh 2006).

NF‐κB subunits, which form homo‐ and hetero‐dimers, are rendered inactive in the cytoplasm by inhibitor of κB proteins (IκB); NF‐κB dimers are translocated into the nucleus because of IκB phosphorylation and degradation brought on by activation of the NF‐κB signaling pathway. These use their Rel homology domain to attach to the promoter regions of the target gene κB. Although p50:p65 is the most well‐known and frequently researched NF‐κB dimer, there are other dimer combinations of NF‐κB with distinct and significant functional outputs as shown in Figure 1B. A transcriptional activation domain (TAD) found in p65, RelB, and c‐Rel allows them to induce gene transcription in their homodimer state (Lawrence 2009; Hoesel and Schmid 2013). Activating NF‐κB results in the transcription of genes that code for growth and angiogenic factors, cell‐cycle modulators, survival signals, and inflammatory cytokines—all of which are essential drivers in an environment that promotes tumor growth. Because of the wide range of proteins that NF‐κB regulates, attempts to block this process with blanket inhibitors of upstream effectors, like IκB Kinase (IKK) inhibitors, have not succeeded, and the off‐target consequences have made matters worse (Gamble et al. 2012). The balance between NF‐κB activation and control is disrupted in pathological circumstances like cancer and chronic inflammation. In this current review, we will discuss a non‐communicable inflammation‐derived disease: cancer. The role of NF‐κB modulation in the management of different cancers by phytochemicals will be discussed in detail.

## 2 Role in Homeostasis

As a key player, NF‐κB governs a person's immunological system dynamics, acting as a critical mediator of both immune regulations (innate and adaptive). In innate immunity, it is rapidly activated in response to PAMPs through TLRs, facilitating the expression of inflammation‐supporting cytokines and antimicrobial peptides that initiate the host defense (Iacobazzi et al. 2023). In adaptive immunity, NF‐κB is indispensable for lymphocytes' ontogenesis, persistence, and operational efficacy, including B and T cells. It regulates the orchestration of gene networks governing antigen receptor signaling, production of cytokines, and lymphocyte proliferation, ensuring a robust immune response to infections. NF‐κB also contributes to maintaining immune tolerance by regulating the differentiation and function of regulatory T cells (Tregs), which prevent autoimmune reactions (Milkova et al. 2010). However, dysregulation of NF‐κB activity can lead to chronic inflammation, autoimmune diseases, and immunodeficiency, underscoring the importance of its precise control in immune homeostasis (Murphy et al. 2022).

NF‐κB exerts a biphasic influence on cellular dynamics by orchestrating mitotic progression and programmed cell death, positioning it as a pivotal regulator of cellular equilibrium and safeguarding against pathologies like malignancies. On one hand, NF‐κB promotes clonal expansion by negatively downregulating the translation products of genes encoding cyclins, CDKs, and growth factors, which drive the cell cycle and support cellular growth (Li et al. 2019). On the other hand, NF‐κB exerts anti‐apoptotic effects by inducing the transcription of survival genes, which cancel out pro‐apoptotic signals and enhance cell survival under stress conditions (Khoshnan et al. 2000). Dysregulation of NF‐κB's role can lead to pathological alterations in cellular propagation and apoptotic regulation, which are prevalent in neoplastic disorders. Its constitutive activation supports tumor growth, cell death resistance, and immune surveillance evasion. Therefore, understanding these molecular mechanisms is critical for the development of targeted therapeutic interventions against cancer and other proliferative disorders.

NF‐κB plays a fundamental role in modulating cellular reactions to oxidative stress due to a disruption in the equilibrium between reactive oxygen species (ROS) accumulation and the body's antioxidant safeguarding systems (Jin et al. 2024). Under oxidative stress conditions, NF‐κB is activated by ROS through mechanisms involving the IKK complex or direct modification of NF‐κB subunits, such as p65, by redox‐sensitive modifications (Ungvari et al. 2011). Once activated, NF‐κB induces the expression of antioxidant genes, including those encoding superoxide dismutase (SOD), glutathione peroxidase (GPx), and catalase, which help neutralize ROS and restore cellular redox balance (Cheleschi et al. 2022). Additionally, NF‐κB regulates gene expression in DNA repair, cell survival, and inflammation, providing a comprehensive defense mechanism against oxidative damage. However, chronic or excessive NF‐κB activation under persistent oxidative stress can cause a cascade of deleterious effects, such as sustained inflammation, tissue damage, and the promotion of diseases like cancer (Romero et al. 2022). Thus, NF‐κB's role in oxidative stress response is double‐functional, highlighting the need for precise regulation to maintain cellular homeostasis.

Tissue repair and regeneration are also areas in which NF‐κB plays a pivotal role. It does this by orchestrating inflammatory responses, cell proliferation, and extracellular matrix remodeling, essential for restoring tissue integrity after injury. Following tissue damage, NF‐κB is activated by damage‐associated molecular patterns (DAMPs) and cytokines, such as TNF‐α and IL‐1β, to recruit immune cells and produce growth factors that promote healing (Palumbo et al. 2007). It controls the transcription of genes associated with neovascularization, including vascular endothelial growth factor (VEGF, which aids in the formation of blood vessels), and extracellular matrix components, such as fibronectin and collagen, which are critical for tissue remodeling. Plus, NF‐κB supports the survival and proliferation of stem and progenitor cells, essential for regenerating damaged tissues (Rinkenbaugh and Baldwin 2016). However, dysregulated or prolonged NF‐κB activation can lead to excessive inflammation, fibrosis, and impaired tissue regeneration, contributing to chronic wounds and pathological scarring (Bannon et al. 2015). Thus, NF‐κB's role in tissue repair and regeneration is tightly regulated to balance inflammation and healing. This makes it a potential target for therapies to enhance tissue recovery.

## 3 The Canonical Versus Non‐Canonical Pathway

NF‐κB could proceed in two ways. The first is a canonical pathway, while the second is a non‐canonical pathway. The canonical path is a highly regulated signaling cascade. This pathway is initially activated when certain immunological factors, including pro‐inflammatory cytokines (such as TNF‐α and IL‐1β), along with pathogen‐associated molecular patterns (PAMPs) (Perkins 2007). Activation begins with the engagement of signal‐receiving factors, which are found on the surface of a cell. The TNF receptor (TNFR) activates this process by recruiting adaptor proteins such as TRADD or MyD88. Such reactions come with causative responses. As a result of this, a complex of macromolecules responsible for the activation of IkB kinase (IKK), another set of complexes constituting IKKα, IKKβ, and the NF‐κB essential modulator (NEMO), gets activated (Karin and Ben‐Neriah 2000). The IKK complex begins phosphorylating IκBα, marking it for the post‐translational modification (ubiquitination) and subsequent proteasomal degradation (Pavithra et al. 2024). This degradation releases the p65/p50 heterodimer, the most common NF‐κB dimer, allowing it to translocate to the nucleus and bind to κB sites in the promoter regions of target genes (Hayden and Ghosh 2012). These target genes encode proteins involved in inflammation, immune response, cell survival, and proliferation, highlighting the pathway's critical role in physiological and pathological processes (Ghosh and Karin 2002). This route of the NF‐κB pathway is essential as it has been frequently observed to malfunction during chronic inflammatory diseases or in various types of cancers. This malfunctioning deviates NF‐κB from normal functioning (Taniguchi and Karin 2018).

**FIGURE 2:**
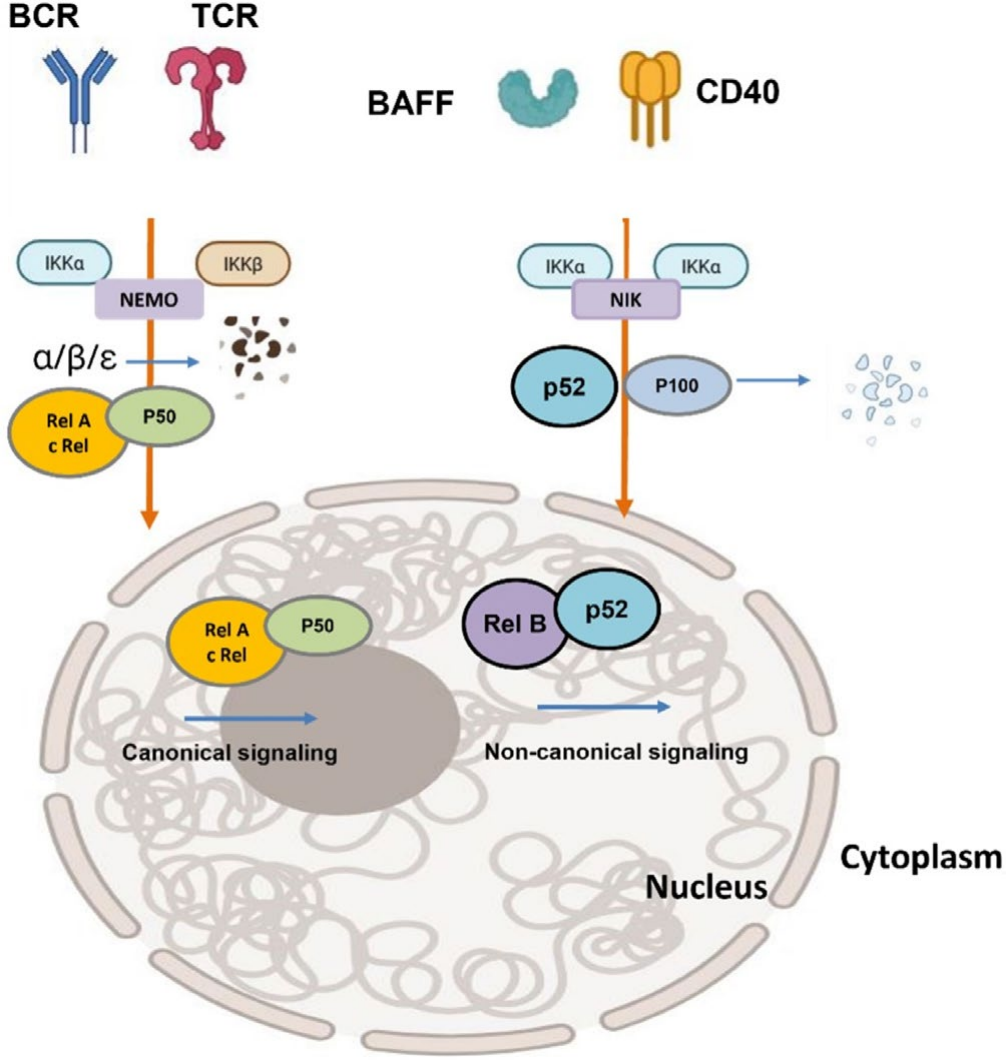
The canonical and non‐canonical NF‐κB signaling pathways are summarized schematically. It is demonstrated that the primary mechanism by which the canonical signaling pathway generates RelA:P50 and cRel:P50 is the degradation of inhibitors of NF‐κB (IκBs). RelB:P52 is activated mainly by the non‐canonical pathway when p100 is processed into p52.

The non‐canonical NF‐κB pathway, on the other hand, is a distinct signaling cascade, critical for immune cell development and lymphoid organogenesis (Perkins 2007). Figure 2 shows the canonical and non‐canonical pathways. Unlike the canonical pathway, this pathway relies on processing the NF‐κB2 precursor protein p100 into its active form, p52, through a tightly regulated mechanism involving NF‐κB‐inducing kinase (NIK) and IKKα (Karin and Ben‐Neriah 2000). In unstimulated cells, NIK is constitutively degraded, keeping the pathway inactive. Upon receptor engagement, NIK stabilization occurs, leading to the phosphorylation and activation of IKKα, which subsequently phosphorylates p100. This phosphorylation triggers the ubiquitination and partial proteasomal degradation of p100, generating the p52/RelB heterodimer (Pavithra et al. 2024) as shown in Figure 1. The p52/RelB complex then translocates to the nucleus, regulating genes involved in B cell maturation, osteoclastogenesis, and secondary lymphoid organ development (Hayden and Ghosh 2012). The non‐canonical pathway is essential for adaptive immunity and has been implicated in autoimmune diseases and certain cancers, particularly those involving B‐cell malignancies (Taniguchi and Karin 2018). Like the canonical route, the non‐canonical NF‐κB pathway is also essential, as its selective activation and regulation make it a promising target for therapeutic interventions in immune‐related disorders.

NF‐κB pathway activation involves multiple stimuli. Upon activation, NF‐κB dimers, such as p65/p50 or p52/RelB, translocate to the nucleus and bind to specific DNA sequences within the regions of target genes (Kim et al. 2023). The transcriptional activity of this pathway is further modulated by interactions with coactivators, corepressors, and chromatin‐remodeling complexes, which influence the accessibility of DNA and the recruitment of RNA polymerase II (Kim et al. 2012). Of various types of post‐translational modifications, ubiquitination, acetylation, and phosphorylation are the major ones that are also critical players for the fine‐tuning of NF‐κB's ability to activate or repress gene expression. For instance, if the protein, p65, undergoes a phosphorylation reaction at specific serine residues, its transcriptional activity gets enhanced. At the same time, acetylation, on the other hand, could have both effects, promoting or limiting, depending on the background (Wang et al. 2000). Also, NF‐κB has been shown to interact with many other transcription factors and signaling pathways, creating a network of regulatory mechanisms that ensure precise and context‐dependent gene expression. To maintain cellular homeostasis and prevent excessive inflammation or linked immune responses, limiting the NF‐κB pathway is necessary. Thus, negative regulation is required. This is essential for avoiding disease‐causing pathological conditions (Perkins 2007).

One of the primary mechanisms of negative regulation involves the inhibitory IκB proteins. These proteins, by their mechanism, inhibit the unrestricted mobility of NF‐κB inside the nucleus, limiting its binding to the cell's DNA. IκB proteins are degraded upon activation, but their rapid resynthesis creates a self‐regulating cycle that curtails the prolonged NF‐κB response and function (Karin and Ben‐Neriah 2000). Additionally, deubiquitinating enzymes (DUBs) such as A20 and CYLD can reverse the ubiquitination of key signaling molecules, including TRAF6 and RIP1, dampening NF‐κB stimulation (Hayden and Ghosh 2012).

## 4 NF‐κB in Different Cancers

**FIGURE 3:**
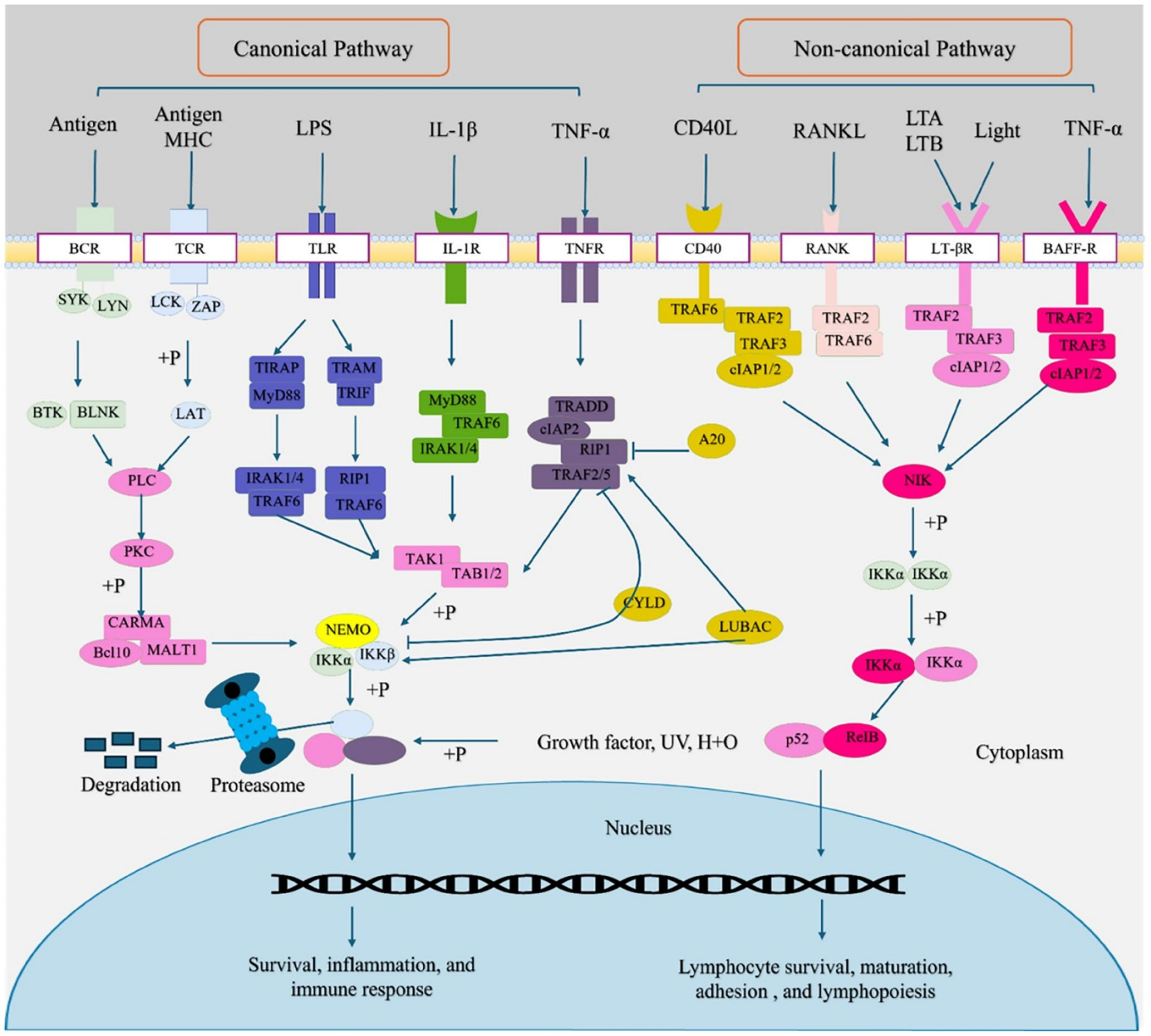
Illustration of canonical and non‐canonical NF‐κB signaling pathways showing receptor activation by antigens, cytokines, and ligands, recruitment of adaptor proteins, kinase cascades, and IκB degradation. The canonical pathway activates p50 RelA dimers, while the non‐canonical pathway involves NIK and p52 RelB processing. Both regulate inflammation, immunity, cell survival, and lymphocyte development and homeostasis.

NF‐κB is a central player in tumorigenesis, driving the initiation and progression of various cancers by regulating genes involved in inflammation, cell survival, and proliferation. Chronic activation of NF‐κB, often induced by persistent inflammatory signals, genetic mutations, or oncogenic stimuli, contributes to transforming normal cells into malignant ones by promoting genomic instability and resistance to apoptosis (Czauderna et al. 2019). NF‐κB enhances the expression of pro‐inflammatory cytokines, such as IL‐6 and TNF‐α, which create a tumor‐promoting microenvironment by stimulating cell proliferation and suppressing immune surveillance (Salah et al. 2023). Additionally, NF‐κB upregulates the transcription of genes encoding cyclins and growth factors, which drive uncontrolled cell cycle progression and tumor growth. Its role in tumorigenesis is further supported by its ability to inhibit apoptosis through the induction of anti‐apoptotic proteins like Bcl‐2 and survivin, allowing cancer cells to evade programmed cell death (Qiao et al. 2013). These mechanisms highlight NF‐κB as a critical factor in cancer development, making it a promising target for therapeutic interventions to disrupt tumorigenic processes (Figure 3).

NF‐κB is a key regulator of cancer cell survival, enabling tumor cells to evade apoptosis and thrive in hostile microenvironments. It promotes survival by upregulating the expression of anti‐apoptotic genes, such as Bcl‐2, Bcl‐xL, and IAPs, which counteract pro‐apoptotic signals and protect cancer cells from programmed cell death (Czauderna et al. 2019). Additionally, NF‐κB enhances the expression of survival factors like survivin and FLIP, which further inhibit caspase activation and apoptotic pathways (Cui et al. 2017). NF‐κB also contributes to cancer cell survival by inducing the production of cytokines and growth factors, such as IL‐6 and IGF‐1, which create autocrine and paracrine signaling loops that sustain proliferation and survival (Cui et al. 2017). Furthermore, NF‐κB activation helps cancer cells adapt to stress conditions, such as hypoxia and nutrient deprivation, by modulating metabolic pathways and enhancing resistance to oxidative stress (Cui et al. 2017). These mechanisms collectively enable cancer cells to survive and proliferate despite therapeutic interventions, underscoring NF‐κB's role as a critical target for strategies aimed at inducing cancer cell death and improving treatment outcomes.

NF‐κB plays a critical role in angiogenesis and metastasis, two hallmarks of cancer progression that enable tumors to grow and spread to distant sites. In angiogenesis, NF‐κB induces the expression of pro‐angiogenic factors, such as VEGF and interleukin‐8 (IL‐8), which stimulate the formation of new blood vessels to supply oxygen and nutrients to growing tumors (Zhang et al. 2018). This process is further supported by NF‐κB's ability to enhance the production of matrix metalloproteinases (MMPs), which degrade the extracellular matrix (ECM) and facilitate endothelial cell migration and vessel formation (Bai et al. 2024). In metastasis, NF‐κB promotes the epithelial‐to‐mesenchymal transition (EMT), a process that enables cancer cells to acquire migratory and invasive properties. It upregulates EMT‐inducing transcription factors, such as Snail and Twist, while downregulating epithelial markers like E‐cadherin (Pavitra et al. 2023). Additionally, NF‐κB enhances the survival of circulating tumor cells by activating anti‐apoptotic pathways, allowing them to withstand the stresses of the bloodstream and colonize distant organs (Huntington et al. 2023). These mechanisms highlight NF‐κB as a central driver of tumor vascularization and dissemination, making it a promising target for therapies to inhibit cancer progression and metastasis.

**FIGURE 4:**
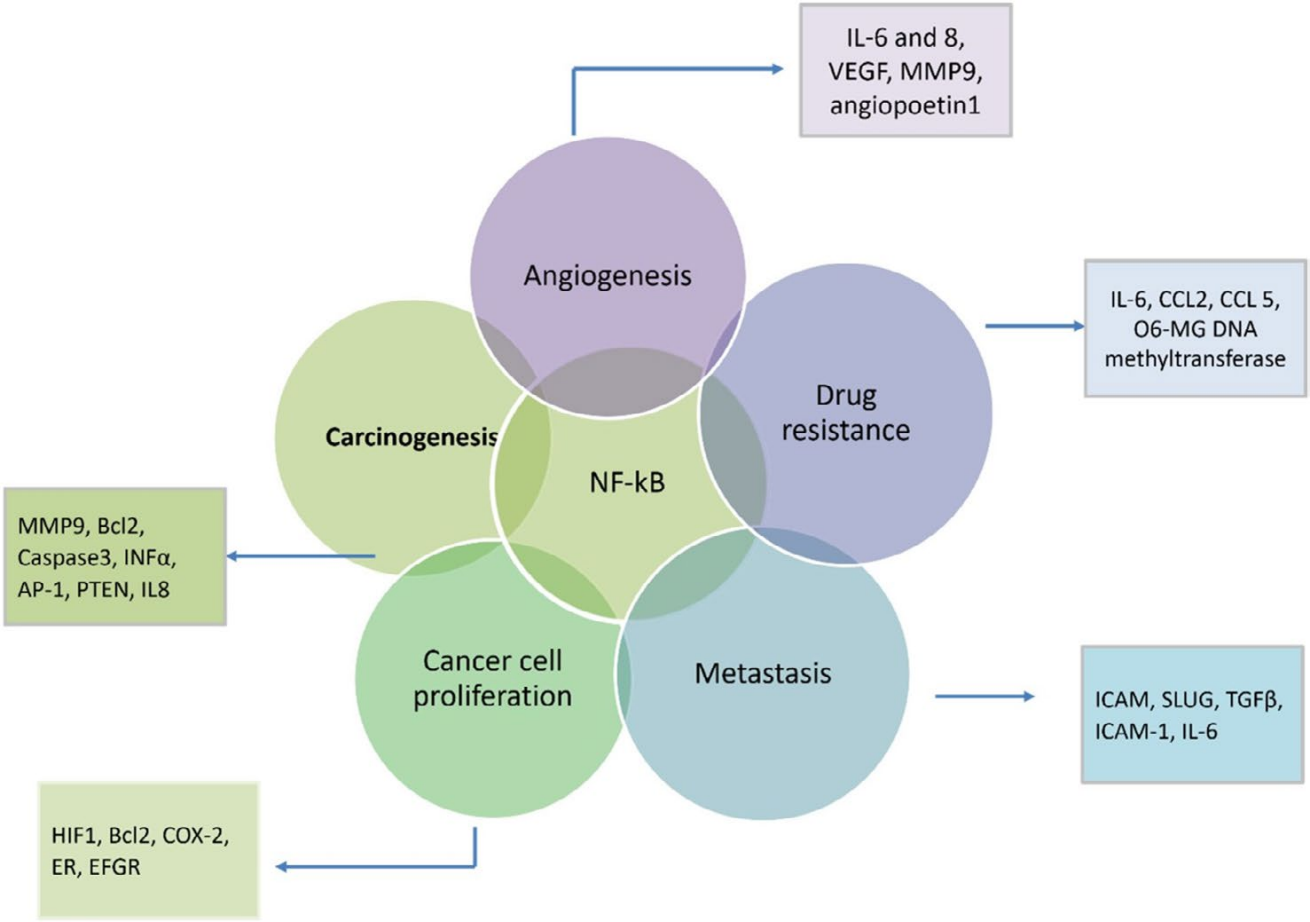
Role of NF‐κB in Cancer Progression. This diagram illustrates the central role of NF‐κB in promoting multiple cancer‐related processes, including carcinogenesis, cancer cell proliferation, angiogenesis, metastasis, and drug resistance. Each process is associated with key molecular targets regulated by NF‐κB, highlighting NF‐κB's contribution to tumor development, survival, invasion, and therapy evasion.

NF‐κB is also a major contributor to chemoresistance, a significant challenge in cancer treatment that limits the efficacy of chemotherapy and leads to disease recurrence (Jiang et al. 2022). NF‐κB activation in cancer cells promotes resistance to chemotherapeutic agents by upregulating anti‐apoptotic protein expression, which protects cells from drug‐induced apoptosis (Chen et al. 2024). Additionally, NF‐κB enhances the expression of drug efflux pumps, such as multidrug resistance protein 1 (MDR1) and breast cancer resistance protein (BCRP), which reduce intracellular concentrations of chemotherapeutic drugs and diminish their effectiveness (Wu et al. 2023). NF‐κB also contributes to chemoresistance by promoting DNA repair mechanisms through the upregulation of genes involved in damage repair, such as XRCC1 and RAD51, allowing cancer cells to recover from chemotherapy‐induced DNA damage (Zheng 2017). Furthermore, NF‐κB‐mediated induction of pro‐survival cytokines and growth factors, such as IL‐6 and IGF‐1, creates a protective microenvironment that supports cancer cell survival despite therapeutic interventions (Makhov et al. 2018). These mechanisms underscore the critical role of NF‐κB in chemoresistance and highlight the need for targeted strategies to inhibit NF‐κB signaling to improve cancer therapy outcomes. Figure 4 summarizes the role of NF‐κB signaling in promoting cancer and various factors associated.

### 4.1 NF‐κB Signaling in Breast Cancer

In women, it is one of the most commonly occurring types of cancer and the second leading type of cancer. In terms of death statistics, this outranks every disease in women. Gene expression regulators, including NF‐κB signaling, are behind the cause of those faulty genes, which later become the cause of these cancers. In this case, our topic of discussion (NF‐κB) is an immune‐stimulatory regulator that triggers multiple tumor‐supporting factors. Ultimately, the expression of such contributors critically modulates various immune responses; it has been predicted that several gene expression regulators, including NF‐κB signaling, are behind the cause of those faulty genes, which later become modulatory genes that aid in the development and nourishment of breast cancer (Pavitra et al. 2023). Breast cancer has several functions encompassing inflammatory responses, clonal amplification, cellular endurance, and others (Niederberger and Geisslinger 2010; Chen et al. 2023; Lin et al. 2023; Qian et al. 2023; Que et al. 2023).

The growth capacity of breast cancer cells is increased by an increase in NF‐κB signaling, which also makes it easier for tumors to move to the liver, lungs, lymph nodes, and bone. This review focuses on how NF‐κB signaling is used by radiotherapy, endocrine therapy, chemotherapy, and targeted therapy to cause breast cancer resistance (Guo et al. 2024). Studies on breast cancer point to a correlation between elevated nuclear localization and NF‐kB 1 expression. A subset of high‐risk breast tumors that are estrogen receptor‐positive have been found to have increased p50 DNA binding and, to a lesser extent, p65 binding (Zhou et al. 2005). Additionally, NF‐kB 1 knockdown in the inflammatory breast cancer cell line (SUM‐149) indicates that NF‐kB 1 expression positively controls cell motility and Rho C expression, which may be a factor in the inflammatory breast cancer's spreading phenotype (Brenner et al. 2008).

### 4.2 Gastric Cancer

When the cancer occurs in the stomach region of the body and starts affecting the digestive system, this cancer type is called gastric cancer (GC) (Chaithongyot et al. 2021). The classical and non‐canonical signaling routes are the two primary signaling pathways that activate NF‐κB dimers. Numerous triggers, including interleukin 1β (IL‐1β), tumor necrosis factor (TNF), and ligands of bacterial origin, can activate the traditional NF‐κB pathway (Napetschnig and Wu 2013). Chronic infections, obesity, and poor diet are the main causes of gastric cancer. *Helicobacter pylori* (*H. pylori*) *is* the primary cause of infection‐driven stomach malignancies. Numerous virulence factors produced by *H. pylori* can activate the NF‐κB pathway, which in turn attracts inflammatory cells and creates inflammatory mediators that promote carcinogenesis (Sokolova and Naumann 2017). It's interesting to note that ADP‐glycero‐β‐D‐manno‐heptose (ADP‐hep), a crucial metabolic step in the formation of lipopolysaccharide (LPS), has been shown to activate NF‐κB in pathogen infection (Pfannkuch et al. 2019). Important components in response to ADP‐hep that activate classical NF‐κB in pathogen infection, including *H. pylori* ‐infected gastric epithelial cells, are the protein alpha‐kinase 1 (ALPK1) and tumor necrosis factor receptor‐associated factor (TRAF)‐interacting protein with fork head‐associated domain (TIFA). Moreover, TRAF6, transforming growth factor β kinase 1 (TAK1), and the IKK complex are involved in *H. pylori* classical NF‐κB activation (Sokolova et al. 2014; Zhou et al. 2018; Pfannkuch et al. 2019).

Polymorphisms in NF‐κB genes and the signaling molecules they produce have been strongly connected to the development of GC. Through regulation of the NF‐κB pathway, the oncoproteins and cellular regulators listed in Table 1 have been implicated in the progression of gastric cancer (GC). All these results highlight NF‐κB as a key contributor to gastric carcinogenesis and a potential target for treatment and prognosis.

**TABLE 1:** Key oncoproteins and cellular regulators involved in gastric cancer progression through modulation of the NF‐κB signaling pathway.

Category	Gene/protein/variant	Key findings	References
NF‐κB gene polymorphisms	*NF‐ΚB 1* (rs28362491, rs230521, rs4648068)	Associated with GC risk and diffuse type; rs28362491 linked to severe gastric inflammation in *H. pylori* ‐infected individuals	(Senol Tuncay et al. 2010; Hua et al. 2014; Chen et al. 2015)
	rs4648068 (A > G)	GG genotype increases transcriptional activity and GC risk in Han Chinese	(Ishikawa et al. 1997; Burkitt et al. 2013)
	*NF‐ΚB 1* deficiency	Causes invasive intestinal‐type GC in mice	(O'Reilly et al. 2018)
	*NF‐ΚB 2* deletion	Leads to gastric hyperplasia and early death in mice; downregulated in GC	(Ishikawa et al. 1997; Low et al. 2020)
	*NF‐κB 1−/−* vs. *NF‐κB 2−/−* mice	*NF‐κB 1−/−* = severe atrophy; *NF‐κB 2−/−* = protected from mucosal lesions	(Burkitt et al. 2013)
	miR‐9	Targets NF‐κB1, regulates GC cell growth	(Wan et al. 2010)
Signaling molecule polymorphisms	*NF‐ΚB IA* (rs2233408, rs17103265, rs696, rs2233406)	Affect GC risk depending on cancer type/location	(Wang et al. 2010; Li et al. 2017)
	*IKBKB* (rs2272736)	A allele associated with better survival, reduced GC mortality	(Gong et al. 2020)
	*TNIP1* (rs7708392, rs10036748)	Increased GC risk in Han Chinese	(Liu et al. 2016)
	*MYD88* (wild‐type and L265P mutant)	Overexpression linked to advanced GC; L265P activates NF‐κB, STAT3, AP1 in MALT lymphoma	(Ngo et al. 2011; Yue et al. 2017)
	*RIPK2* (rs16900627)	Upregulated in GC; minor allele increases intestinal‐type GC risk	(Yang, Tian, et al. 2021)
	*TLR9‐1237 T/C*	Enhances premalignant changes in *H. pylori* ‐infected mucosa via NF‐κB activation	(Pimentel‐Nunes et al. 2013; Wang, Peng, et al. 2014)
Functional and molecular modulators	NF‐κB activation (RelA)	Correlates with GC invasion and tumor size	(Sasaki et al. 2001)
	NF‐κB1/RelA knockdown	Inhibits GC invasion, migration, and tumor growth in xenografts	(Huang et al. 2016)
	*MTDH* and miR‐3664‐5P	MTDH activates NF‐κB; miR‐3664‐5P suppresses GC progression via MTDH inhibition	(Li et al. 2014; Jiao et al. 2019)
	*CARD6*	Overexpressed in GC, promotes inflammation	(Kim et al. 2010)
	*PRL‐3*	Promotes GC invasion via NF‐κB–HIF‐1α–miR‐210 axis	(Zhang et al. 2016)
	Other Modulators Modulate GC progression through NF‐κB signaling	Cullin 4A, TNF, GKN1, IL‐17A, IL‐1β, radixin, Fn14, ING4, TFF1, CTGF, CEACAM19, Ku, MT2A, SIRT1, LMP1/LMP2A, microRNAs, spermine oxidase	(Lee et al. 2007; Canedo et al. 2008; Mao et al. 2011; Soutto et al. 2011; Wang, Wu, et al. 2014; Xing et al. 2015; Zhu et al. 2016; Gong et al. 2017)

### 4.3 Prostate Cancer

In prostate cancer (PCa), NF‐κB signaling upregulates androgen receptor (AR) splice variants. This aids the tumor‐supporting processes, which are resilient even to surgical removals of testicles (Lessard et al. 2003). Androgens are sex hormones that control the growth and maintenance of male characteristics and aid in the start of puberty and the preservation of female reproductive health. Androstenediol, testosterone, and dihydrotestosterone (DHT) are potent androgens produced in the δ‐4 steroidogenic pathway that have a high affinity for the androgen receptor (AR) (Guarneri and Kamboj 2019; Elzenaty et al. 2022). Early‐stage and castration‐sensitive PCa (CRPC) growth is exclusively mediated by androgen signaling pathways.

As a regulator of AR, NF‐κB can affect how CRPC develops and progresses. When AR is activated, the potential for PCa to proliferate is significantly increased. To accelerate the advancement of PCa, NF‐κB increases AR expression (Zhang et al. 2009). By attracting cytokines that promote tumor growth, NF‐κB accelerates the course of CRPC. NSD2 hyperactivates NF‐κB in CRPC, which in turn boosts cytokine levels of IL‐6, IL‐8, and TNF‐α, hastening the course of CRPC. To promote CRPC malignancy, cytokines such as IL‐6, IL‐8, and TNF‐α trigger NF‐κB (Yang et al. 2012). Upstream mediators can accelerate PCa progression by activating NF‐κB. By controlling NF‐κB, HMGB1 upregulation promotes carcinogenesis. Upregulation of HMGB1 accelerates the development of PCa and increases TNFR1 expression, which triggers NF‐κB and causes PCa malignancy (Jung et al. 2021). NF‐κB is crucial in accelerating growth and intensifying EMT by suppressing E‐cadherin. Additionally, hypoxia induces overexpression of HIF‐1 and NF‐κB, elevating CXCR3 levels and promoting carcinogenesis (Ashrafizadeh et al. 2020).

### 4.4 Colorectal Cancer

Colorectal cancer is a severe form of cancer that occurs in the rectum and affects both men and women. However, it is more likely to develop in men than women of the same age group (Abancens et al. 2020). NF‐κB here mediates cancer progression through its close association with inflammation‐linked events. These events are triggered by certain molecular factors within the body, such as the COX‐2 gene and inflammation‐supporting cytokines in intestinal cells. Thus, it allows the formation of an environment that favors cancer formation and survival (Jänne and Mayer 2000).

Furthermore, it has recently been reported that p50 homodimers disrupt the polarization of macrophage M1, which contributes to the development of colorectal cancer. NF‐κB 1−/− mice showed higher production of IL‐12 and CXCL‐10 and fewer colorectal cancers in a study, which supports the theory that p50 homodimers inhibit these genes in macrophages, coordinating their pro‐tumorigenic behavior. The anti‐apoptotic protein BAG‐1 (Bcl‐2‐associated athanogene) is abundantly expressed in colon cancer and pre‐malignant tissue. Both colorectal epithelial cells and the colorectal cancer cell line HCT116 have shown the interaction between BAG‐1 and p50 homodimers, with cell death resulting from suppression of either protein. Both the EGFR and the COX‐2 gene (PTGS2) promoters exhibit this complex. Additionally, the complex suppresses EGFR transcription and promotes COX‐2 transcription, both of which are known to promote colon cancer, allowing it to differentially control gene expression.

### 4.5 Lung Cancer

NF‐κB is highly expressed in lung cancer; predominantly, the non‐small cell lung cancer (NSCLC). As RelA has been linked to maturation during lung morphogenesis through modification of the apoptotic processes of lung epithelial cells, NF‐κB is an important signaling mechanism for the respiratory system (Londhe et al. 2008). Furthermore, by blocking NF‐κB constitutive activation, it has been shown that NF‐κB directly regulates VEGFR2, which is essential for pulmonary angiogenesis and alveolarization during postnatal development (Chen et al. 2011). The expression of all NF‐κB subunits was higher in tumor tissue than in normal lung. The most common is RelB, however, normal bronchial and alveolar epithelial cells that have had tumors removed also have RelA, p50, and p52. The tissues surrounding the tumor likewise showed elevated expression of all NF‐κB subunits. RelA, RelB, and p50 were more common than p52 in tumor tissues and tumor stroma, although NSCLC tissues from various pathologies did not express distinct NF‐κB subunits differently (Giopanou et al. 2015).

Furthermore, elevated NF‐κB expression in individuals with lymph node metastases and late‐stage NSCLC suggested that this disease had some tumor‐promoting function. The correlation between NF‐κB expression and 5‐year OS may be related to its subcellular localization, as expression of NF‐κB in the nucleus (but not the cytoplasm) has been linked to a worse 5‐year OS for NSCLC (Gu et al. 2018; Zhang et al. 2023).

### 4.6 Glioblastoma Multiforme (GBM)

Tumor makes life difficult, especially in the brain, as they may interfere with normal thinking processes and daily physical activities. GBM is one such kind that makes up more than half of all brain‐reported tumors, with worldwide detection of about three to four cases out of every hundred thousand individuals each year (Saeed et al. 2025). While it is fatal, another major problem with it is its detection, as it could be caused due to various factors, including genetic alterations, NF‐κB signaling modification, epigenetic alterations, environmental, and lifestyle factors (Friedmann‐Morvinski et al. 2016; Saeed et al. 2025). To identify the role of NF‐κB signaling, Friedmann‐Morvinski et al. (2016) conducted research and concluded that limiting its functioning or, on the other hand, silencing Timp1 could decrease the speed of progression of GBM (Saeed et al. 2025). Robe et al. (2004) have shown that sulfasalazine is a potential compound which, in their study, was able to reduce GBM‐linked cells via apoptosis triggered by it (Robe et al. 2004). Involvement of NF‐κB in GBM was confirmed by Gill et al. (2002); they also developed an idea to counteract it with the usage of a decoy oligonucleotide, which resulted in cell number (Gill et al. 2002).

### 4.7 Other Cancer Types

Just like these mentioned cancer types, NF‐κB has roles in various other types of cancer too, through various mechanisms. NF‐κB, for instance, could lead to the expression and maintenance of carcinogens or factors which could cause lung cancer, leukemia, multiple myeloma, head and neck cancer, and ovarian cancer, etc. It triggers specific molecular events in each type and helps tumor cells grow or evade the defense mechanisms that could eliminate them in a healthy person.

## 5 Phytochemicals as Modulators of NK‐κB Signaling

Phytochemicals, naturally occurring compounds derived from plants, exert their anti‐cancer effects through diverse mechanisms that target multiple signaling pathways in tumorigenesis (Anwar, DasGupta, Azum, et al. 2022; Adnan et al. 2023; Jang and Lee 2023). These bioactive compounds modulate key cellular processes, including inflammation, oxidative stress, apoptosis, and cell cycle regulation, by interacting with molecular targets such as transcription factors, kinases, and enzymes. For instance, phytochemicals can inhibit the activation of NF‐κB, which in this literature has already been mentioned as a central regulator of inflammation and cancer progression, by blocking upstream signaling molecules like IKK or preventing the degradation of IκBα. Additionally, they could enhance the expression of antioxidant enzymes, like SOD and GPx, to counteract oxidative stress and reduce DNA damage (Cheng et al. 2017). Phytochemicals also induce apoptosis in cancer cells by activating intrinsic and extrinsic apoptotic pathways while inhibiting anti‐apoptotic proteins like Bcl‐2 and survivin (Cheng et al. 2017). Furthermore, they regulate the cell cycle by modulating the expression of various other associated proteins, leading to cell cycle arrest and inhibition of tumor proliferation (Murad et al. 2016). These multifaceted mechanisms highlight the potential of phytochemicals as versatile agents for cancer prevention and therapy.

Dietary polyphenols have emerged as potent inhibitors of NF‐κB activation, offering a promising strategy to disrupt the signaling pathways that drive inflammation and cancer progression. These natural compounds target various steps in the NF‐κB signaling cascade, including the inhibition of upstream kinases like IKK, which prevents the phosphorylation and degradation of IκBα, thereby retaining NF‐κB in the cytoplasm. For example, curcumin, a polyphenol derived from turmeric, directly inhibits IKK activity and suppresses the nuclear translocation of NF‐κB dimers, reducing the expression of pro‐inflammatory and pro‐survival genes (Kasinski et al. 2008). Similarly, resveratrol, found in grapes and red wine, blocks NF‐κB activation by inhibiting the phosphorylation of IκBα and reducing the DNA‐binding ability of NF‐κB. Other phytochemicals, such as epigallocatechin gallate (EGCG) from green tea, modulate NF‐κB signaling by targeting ROS‐mediated pathways, which are often involved in NF‐κB activation (Sharifi‐Rad et al. 2020). By suppressing NF‐κB activation, these phytochemicals not only reduce inflammation but also inhibit tumor cell survival, proliferation, and metastasis, making them valuable candidates for cancer prevention and therapy.

Dietary polyphenols also significantly mitigate inflammation and oxidative stress, two interconnected processes that contribute to the development and progression of chronic diseases, including cancer (Kang et al. 2022). These natural compounds exert anti‐inflammatory effects by inhibiting the activation of NF‐κB, a master regulator of inflammatory responses, thereby reducing the production of pro‐inflammatory cytokines such as TNF‐α, IL‐6, and IL‐1β (Kim et al. 2024). Dietary polyphenols such as quercetin are particularly effective in neutralizing ROS and reducing oxidative damage to DNA, lipids, and proteins, thereby protecting cells from stress‐induced apoptosis and mutations. By simultaneously targeting inflammation and oxidative stress, dietary polyphenols provide a dual protective mechanism that helps maintain cellular homeostasis and prevents the initiation and progression of cancer (Tabolacci et al. 2019).

Dietary polyphenols profoundly affect apoptosis and cell cycle regulation, making them valuable agents in cancer prevention and therapy (Kesavan et al. 2023). These compounds induce apoptosis in cancer cells by activating both intrinsic and extrinsic apoptotic pathways. For instance, curcumin and resveratrol upregulate pro‐apoptotic proteins such as Bax and Bak while downregulating anti‐apoptotic proteins like Bcl‐2 and Bcl‐xL, leading to mitochondrial outer membrane permeabilization and caspase activation (Golestannezhad et al. 2024). Additionallyas the polyphenols canstimulate the extrinsic pathway by enhancing the expression of death receptors like Fas and TRAIL, which initiate caspase‐8‐mediated apoptosis (Wani et al. 2023). In terms of cell cycle regulation, polyphenols modulate the expression of cyclins, CDKs, and CDK inhibitors, leading to cell cycle arrest at specific checkpoints. For example, curcumin induces G2/M phase arrest by downregulating cyclin B1 and CDK1, while EGCG promotes G1 phase arrest by upregulating p21 and p27, inhibitors of CDK4/6 (Jafari et al. 2014). These mechanisms inhibit cancer cell proliferation and confer a selective advantage by sparing normal cells, highlighting the potential of dietary polyphenols as targeted therapeutic agents (Hao et al. 2022). By simultaneously modulating apoptosis and the cell cycle, phytochemicals offer a multifaceted approach to suppressing tumor growth and enhancing the efficacy of conventional cancer treatments (Mahajna et al. 2019).

## 6 NF‐κB‐Targeting Dietary Polyphenols in Cancer Therapy

Dietary polyphenols are plant derived bioactive phytochemicals that modulate NF‐κB signaling, a central node for inflammation and cancer. These proteins arrest NF‐κB activity through either the IκBα degradation or targeting upstream kinases such as IKK. Some of the phytochemicals also inhibit NF‐κB‐associated downregulation of inflammation‐supporting and pro‐survival genes, thereby promoting their growth and therapeutic efficacy against tumors. Since they control oxidative stress, apoptosis, and immune responses, we think this makes them very attractive candidates for cancer prevention and/or treatment (Chauhan et al. 2022).

### 6.1 Polyphenols as NF‐κB inhibitors

**FIGURE 5:**
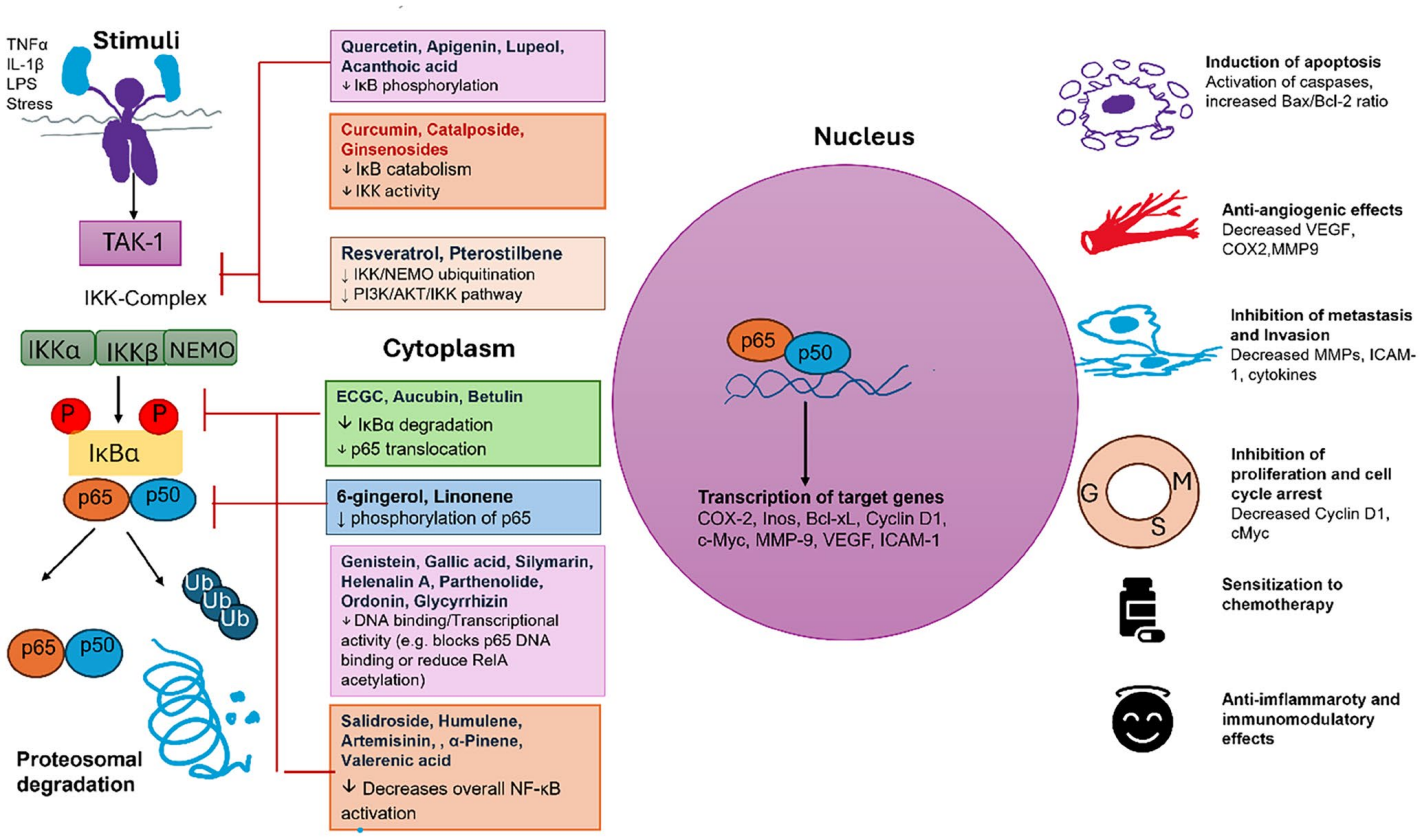
Mechanistic overview of dietary polyphenols and phytochemical‐mediated inhibition of NF‐κB signaling. The schematic illustrates multiple regulatory checkpoints within the canonical NF‐κB pathway targeted by bioactive natural compounds.

Polyphenols are widely occurring natural compounds, abundant in plants and are a part of the human diet. Polyphenols show inhibitory effects against cancer cells via modulating many signaling pathways, including NF‐κB signaling. The natural compounds inhibit kinase phosphorylation, preventing NF‐κB from accessing the nucleus, which blocks its interaction with DNA. NF‐κB inhibition by polyphenols shows various biological effects such as reduction of inflammation, promotion of apoptosis, and therefore preventing cancer progression (Khan et al. 2020). Figure 5 shows the different mechanistic approaches by dietary polyphenols and phytochemicals in inhibiting NF‐κB activity. Flavonoids are natural polyphenolic compounds that have two benzene rings joined together via a heterocyclic pyran or pyrone ring. Flavonoids exert a wide range of biological activities and are a part of dietary polyphenols. Fruits, vegetables, and herbs are enriched with flavonoids such as blueberries, oranges, green tea, red wine, etc (Devi et al. 2009). The group of flavonoids contains a wide range of chemical compounds which show biological activities such as anti‐inflammatory, antioxidant, anti‐angiogenic, anti‐cancer, and many more. It has been proposed that flavonoids target many biological pathways, including the NF‐κB pathway, to show modulatory effects on diseases and human health. Lignans are other subclass of polyphenols classified in the class of secondary metabolites, which are derived from the phenylpropanoid pathway. Lignans play an important role in the protection of plants and are a source of human nutrition. Lignans are present in fruits, vegetables, legumes, oils, and cereals (Landete 2012; Haworth 1942). In this section, we will discuss some of the well‐known dietary polyphenols including other sub‐classes of polyphenols including lignan, stilbene etc. which show anticancer activities by targeting the NF‐κB pathw (Zahra et al. 2024; Duo et al. 2026; Kopustinskiene et al. 2020; Pandey et al. 2025).

#### 6.1.1 Quercetin

Quercetin, a plant‐derived compound that falls under the class of flavonoids, is a dietary polyphenol found in many fruits, vegetables, and other natural food sources (Dahiya et al. 2019). It inhibits NF‐κB induction via the mechanisms of the prevention of IκB phosphorylation and thereby suppresses NF‐κB translocation. It also performs the same by limiting DNA binding (Endale et al. 2013; Yang et al. 2023). Quercetin shows anticancer activities in colon cancer cell lines (CACO‐2 and SW‐620) by inhibiting the NF‐κB signaling pathway with IC_50_ values of 35 μM and 20 μM, respectively. Mechanistically, the compound in colon cancer cell lines inhibits NF‐κB DNA‐binding activity and induces dephosphorylation and up‐regulation of IκB‐α, thereby suppressing NF‐κB activation (Zhang et al. 2015).

Quercetin is also known for inhibiting the growth of non‐small cell lung cancer cells by suppressing the NF‐κB signaling pathway. Mechanistically, it decreases NF‐κB and IKKα expression while increasing IκBα; therefore, hindering growth in lung cancer cells (Youn et al. 2013). The compound exhibits anticancer effects via NF‐κB modulation in osteosarcoma cells. NF‐κB suppression promotes apoptosis and enhances cytotoxicity in osteosarcoma cells. Therefore, quercetin acted as an adjuvant anticancer agent by suppressing NF‐κB and mediating chemotherapy‐induced cancer cell death (Ahmadi et al. 2022).

Quercetin has been studied in a very small number of clinical trials for cancer, and most of them are up to early phases. The trials focused on safety and pharmacokinetics rather than effectiveness in therapeutics. Early phase I studies of quercetin administration in patients with advanced malignancies showed safety in administration as well as inhibited tumor biomarkers (Ferry et al. 1996). Quercetin may improve treatment tolerance or modify drug resistance when combined with traditional chemotherapy, according to further studies, but the findings are still unclear and not statistically significant (Lamson and Brignall 2000).

#### 6.1.2 Apigenin

Apigenin, a dietary polyphenol classified further as a flavonoid, is present in chamomile and other natural sources. It has potent neuroprotective, anti‐inflammatory, and anti‐cancer effects (Hussain et al. 2025). It interferes with NF‐κB signaling by limiting COX‐2 expression and diminishing the biosynthesis of pro‐inflammatory cytokines (Wang, Peng, et al. 2014). Apigenin mitigates inflammation‐induced tumorigenesis through these mechanisms, contributing to its chemopreventive potential (Hussain et al. 2025). Apigenin shows anti‐proliferative effects in gastric cancer cells in a time and dose‐dependent manner. In a study by Pratas et al., apigenin induced apoptosis and suppressed tumor development by modulating the Akt/Bad/Bax and IκBα/NF‐κB pathways, along with ROS regulation (Pratas et al. 2024).

Apigenin binds directly to IKK, inhibiting its function and further hindering NF‐κB/p65 activation. The natural compound performed better than the conventional IKK inhibitors in prostate cancer cells, leading to cell‐cycle arrest and suppression of tumor growth. Apigenin suppresses NF‐κB signaling in lung cancer cells, leading to antitumor activity by enhancing TRAIL‐induced apoptosis.

#### 6.1.3 Epigallocatechin Gallate

The bioactive compound is a dietary polyphenol and is a predominant catechin found in *Camellia sinesis*, which modulates NF‐κB activity by preventing LPS‐induced IκBα catabolism and inhibiting RelA nucleoplasm migration (Joo et al. 2012). These actions help suppress NF‐κB‐driven inflammatory and survival pathways, reinforcing Epigallocatechin gallate (EGCG's) role in cancer prevention and therapy. NF‐κB activation is suppressed in the presence of EGCG as the compound inhibits the degradation of the NF‐κB repressor (Zhang et al. 2019) thereby blocking the nuclear translocation of p65 or p50 (Zhang et al. 2019) (Yan et al. 2012). Clinical studies on EGCG have primarily focused on safety and supportive treatment rather than direct effects on tumor. A Phase I clinical trial was conducted in patients with lung cancer undergoing radiotherapy, and it demonstrated that EGCG was tolerated well with no toxicity. However, the impact on tumor progression and overall outcome of the effect of EGCG was not significant (Chow et al. 2003; Ghafghazi et al. 2026). EGCG is presently regarded as an experimental adjunct in oncology, with possible advantages, but not enough data to justify its usage as a conventional cancer treatment.

#### 6.1.4 Genistein

The dietary polyphenol genistein, is an isoflavone found in soybeans, modulates NF‐κB signaling by downregulating its production and limiting its DNA‐binding and transcriptional activities (Zhou et al. 2014). This suppression contributes to its anti‐inflammatory and anticancer properties, making genistein a promising candidate for cancer therapy. Genistein inhibits NF‐κB activity in breast cancer cells and inhibits cancer cell growth and metastasis (Mukund 2020; Joshi et al. 2023). Genistein was studied on A2780 and C200 ovarian cancer cell lines, the compound showed decreased NF‐κB and hence inhibition of cancer progression (Solomon et al. 2008) Effect of genistein in human malignant neuroblastoma SK‐N‐DZ and SH‐SY5Y cell lines was investigated, and NF‐κB was among some of the factors that showed reduced activity upon treatment with genistein (Roy Choudhury et al. 2010). Genistein has inhibitory effects on liver cancer, colon cancer, cervical cancer and osteosarcoma via targeting NF‐κB pathway (Liang et al. 2012; Sahin et al. 2012; Luo et al. 2014; Wang, Chen, et al. 2014).

#### 6.1.5 Gingerol

Gingerol, the active component of ginger, exerts anti‐inflammatory and anticancer effects by targeting NF‐κB signaling. Chemically, it is a phenolic compound because it contains a phenolic hydroxyl group, but it is not usually classified as a classical dietary polyphenol. It inhibits TPA‐induced phosphorylation of p65, thereby reducing NF‐κB activation and subsequent inflammatory responses (Kim et al. 2005). This regulation highlights its potential as a natural anti‐cancer agent by limiting tumor‐promoting inflammation. Gingerol inhibits cervical cancer through inhibition of IκBα, which further prevents NF‐κB from entering the nucleus and induces apoptosis (Zivarpour et al. 2021). Some of the studies claim that gingerol with other active compounds of ginger show inhibitory effects on gastrointestinal tumors (Cheng et al. 2023).

#### 6.1.6 Gallic Acid

It is a naturally occurring dietary polyphenol from various natural sources and foods. It has been used for centuries for its beneficial properties, including antioxidant, anti‐inflammatory, and antimicrobial roles. Gallic acid is commonly used as a preservative in food products and as an astringent in cosmetics and offers protection against UV radiation. Gallic acids lower the levels of acetylation of RelA to show its effect in limiting the NF‐κB pathway (Choi et al. 2009).

#### 6.1.7 Pterostilbene

Pterostilbene is a naturally occurring dietary polyphenol in the subclass stilbene that originates from blueberries and exhibits medicinal benefits. Structurally, it is like resveratrol but with superior bioavailability. Published sources also suggest it may help suppress the initiation of the PI3K/Akt/IKK signaling route (Wang and Chen 2014). Pterostilbene shows anticancer activities against various cancer types by inhibition and activating various biological pathways. The compound shows negative correlation with colon cancer and skin cancer survival by inhibiting the NF‐κB pathway (Chiou et al. 2011). Pterostilbene blocks key signaling pathways (PI3K/Akt and PKC) that normally activate NF‐κB and AP‐1 that prevent processes like EMT and MMP‐9 activity, which cancer cells use to move and invade other tissues. It also stops NF‐κB activation through IAP proteins, even without triggering cell death (Varfolomeev et al. 2007; Pan et al. 2009; Chen et al. 2018).

#### 6.1.8 Resveratrol

Grapes and similar plants, such as berries and peanuts, are the source of a well‐documented dietary polyphenol called resveratrol, further subclassified in stilbenes. It is a polyphenolic stilbenoid classified as an immuno‐protective compound obtained naturally from the mentioned plants in response to stress, pathogens, or UV radiation (Salehi et al. 2018). Resveratrol decreases the transcriptional role of the protein p66. Because of this, it can halt covalent ubiquitin modification of various targets which are found in NF‐κB signaling, and without which this pathway cannot proceed. The targets of resveratrol in NF‐κB signaling are NEMO and IKK. It thus ultimately inhibits the NF‐κB pathway (Ren et al. 2013). Resveratrol is a promising compound in pre‐clinical studies; however, its effectiveness in humans is not clear due to low bioavailability and pharmacokinetics. Some limited existing trials suggest resveratrol's effectiveness may differ based on cancer type, stage, dosage, and treatment duration (Kulkarni and Cantó 2015).

#### 6.1.9 Salidroside

Salidroside is a polyphenol and glucoside of tyrosol primarily found in *Rhodiola rosea* and related species (Liang et al. 2024). It serves as a key bioactive compound in traditional medicine, recognized for its adaptogenic properties and diverse drug‐like properties, embodying free radicals limiting, inflammation opposing, anti‐hypoxic, neuroprotective, and reno‐protective properties (Liang et al. 2024). It could be used to modify the NF‐κB, as salidroside inhibits its phosphorylation (Wang, Lu, et al. 2019). In lung cancer cells damaged by bleomycin, salidroside shows protective effects. The compound suppresses the activation of NF‐κB by preventing IκBα phosphorylation and therefore preventing the nuclear translocation of NF‐κB (p65). Additionally, it activates the Nrf2 antioxidant pathway which works as an antioxidant (Tang et al. 2016).

#### 6.1.10 Curcumin

Turmeric is one of the most essential spices in almost every household and is used for its intense yellowish color and nutritional benefits. This spice has some significant properties due to Curcumin, a dietary polyphenol used in traditional medicine for a long time. Curcumin is further classified as a diarylheptanoids. Curcumin inhibits NF‐κB signaling by suppressing the phosphate group addition and catabolism of IκB and its transfer to the nucleus (Buhrmann et al. 2011; Lai et al. 2024).

Curcumin showed inhibition of cervical cancer cells via suppressing NF‐κB signaling pathway. In HeLa cells, curcumin inhibited NF‐κB and Wnt/β‐catenin pathways, resultant in reduced cell growth and invasion (Ghasemi et al. 2019). The natural compound induces inhibition of various cancer types by mechanistically targeting the NF‐κB signaling. Some of the cancer types include breast, bladder, pancreatic, prostate, ovarian cancer and leukemia (Deeb et al. 2004; Sharma et al. 2006; Alaikov et al. 2007; Bachmeier et al. 2007; Kamat et al. 2007; Kunnumakkara et al. 2007; Lin et al. 2007). Curcumin has been evaluated in multiple Phase I and II clinical trials in patients with solid tumors, including lung cancer, and has been shown to be safe and perhaps, to improve treatment‐related results when combined with targeted therapy or chemotherapy. However, more studies are needed to conclude the effectiveness of curcumin in clinical trails (Arslan et al. 2022).

#### 6.1.11 Honokiol

It is a bioactive lignan and polyphenol made from the plant's outer layers, particularly belonging to the *Magnolia* species, notably *Magnolia officinalis*, with demonstrated antitumor, anti‐inflammatory, antimicrobial, and neuroprotective properties. However, the bark is not the only part from which it is obtained. Honokiol could also be obtained from other parts of the same plants, like the leaves and seed cones, making the whole plant an essential natural product. Honokiol is considered a dietary supplement or natural therapeutic agent, rather than a standard dietary food. This lipophilic compound inhibits cancer progression by targeting pathways like NF‐κB and Akt, suppressing angiogenesis, and inducing apoptosis through mitochondrial mechanisms while also exhibiting antioxidant effects and crossing the blood–brain barrier for potential neurological applications (Fried and Arbiser 2009). Traditionally used in Asian medicine, its broad therapeutic potential extends to dermatology, oncology, and immunomodulation, supported by preclinical studies highlighting its safety and bioavailability (Li, Liang, et al. 2022).

#### 6.1.12 Silymarin

Silymarin is a phytochemical complex composed primarily of flavonolignans, including silybin (the most active and abundant component), isosilybin, silychristin, and silydianin (Nawaz et al. 2023). This phytochemical started to become popular because of its medical properties, which include the protection of the liver, free radical scavenger, pro‐inflammatory pathway suppression characteristics, stabilizing liver cell membranes, promoting regeneration, and counteracting toxins. Silymarin blocks the binding crosstalk concerning NF‐κB and the consumer's DNA to modulate the NF‐κB pathway (Saliou et al. 1998).

#### 6.1.13 Carnosol

This phytochemical is a popular bisphenolic abietane‐type diterpenoid naturally occurring in Salvia rosmarinus and *Salvia officinalis* . It is recognized for its oncopreventive and anti‐phlogistic attributes, contributing significantly to the antioxidant activity of these herbs (Johnson 2011). Carnosol limits the NF‐κB signaling by the suppression of IκBα phosphotransferase activity (Chao et al. 2005). Experimentally it has been observed that Carnosol downregulates certain molecular factors that support cancer. It does so by the prevention of TNF‐α driven phosphorylation, as reported in endothelial and certain other cell lines of rat models (Li, Zhang, et al. 2022). Carnosol also inhibits the action of other kinases that are involved in the MAPK pathway; as a result, the expression of NF‐kB is altered (Yao et al. 2014). In multiple models of cancer, it has demonstrated several positive actions which help fight cancer. It thus lowers cell proliferation, migration, and invasion, and makes them susceptible to chemotherapy. All these factors are achieved by reducing NF‐kB's resistance. Because of all such diseases' modifying properties, Carnosol is considered a good candidate to be used as a medicinal target or as an adjunctive property (Li, Zhang, et al. 2022).

## 7 NF‐κB‐Targeting non‐dietary polyphenols and other Phytochemicals in Cancer Therapy

### 7.1 Quinones

Quinones are natural compounds which show promising anti‐cancer potential. They exhibit unique chemical properties and mechanisms of action including apoptosis in cancer cells. Quinones can easily transform into other forms and have an intramolecular unsaturated cyclic diketone structure. The four primary structural subgroups of natural quinones are benzoquinone, naphthoquinone, anthraquinone, and phenanthraquinone (Patel et al. 2021). The unique chemical structure of quinones imparts various biological properties such as anticancer, antibacterial, antifungal, antiviral, and anti‐atopic dermatitis (Faizan et al. 2023).

#### 7.1.1 Plumbagin

This plant‐based compound is most commonly obtained from the naphthoquinone compound from the *Plumbago*, *Droseraceae* and *Ebenaceae* species plants (Petrocelli et al. 2023). Previous research studies have indicated multifaceted roles, which encompass antioxidation, anti‐inflammation, antimicrobial, and anticarcinogenic properties, primarily through mechanisms such as redox cycling, ROS generation, and metal chelation (Yin et al. 2020). The compound also demonstrates efficacy against drug‐resistant bacteria by disrupting efflux pumps and eliminating multidrug‐resistant plasmids (Nair et al. 2016). Its anticancer properties involve apoptosis induction and NF‐κB pathway inhibition (Padhye et al. 2012). Additionally, plumbagin modulates bone metabolism by promoting osteoblast activity and inhibiting osteoclast function (Yadav et al. 2022).

### 7.2 Iridoids

Glycosides called iridoids are present in a variety of plants and are said to bind to glucose. They feature an iridodial‐like molecular structure and the general form of cyclopentopyran. The presence or lack of intramolecular glycosidic linkages determines the structural classification of iridoids into iridoid glycosides and non‐glycosidic iridoids; iridoid glycosides can also be further differentiated into carbocyclic iridoids and secoiridoids. Iri (Kim and Choi 2021). Iridoids exhibit anti‐proliferative activities in cancer cells (Tundis et al. 2008).

#### 7.2.1 Aucubin

Aucubin is an iridoid glycoside found in various plant families. It exhibits a broad spectrum of biological activities, including anti‐inflammatory, antioxidant, and anticancer properties. Aucubin is unstable and can be converted into its aglycone, aucubigenin (Kartini et al. 2023). Preventing IκBα degradation and nuclear translocation of the p65 subunit (Dinda et al. 2007), aucubin could also modulate the NF‐κB signaling pathway. Aucubin is a promising anti‐cancer compound; however, the studies are limited to pre‐clinical studies.

#### 7.2.2 Catalpoloside

Catalpoloside or catalpol is another iridoid compound that is isolated from *Catalpa ovata* . This phytochemical has been studied and shown to have inflammation‐limiting potential by attenuating the expression of inflammatory processes supporting genes, such as TNF‐α‐induced interleukin‐8 (IL‐8) in human intestinal epithelial cells (Kim et al. 2004). Catalposide can inhibit the catabolism of IκBα protein and could drive the nucleocytoplasmic transport of p65 domain (Kim et al. 2004), hence modulating the NF‐κB signaling pathway. The lack of clinical trials on catalpol; the studies are limited to in vitro and in vivo studies focusing on catalpol's anticancer effects. In a study, catalpol inhibited a process (EMT) that mediated lung cancer cells in invasive, by blocking key signaling pathways (Smad2/3 and NF‐κB) activated by TGF‐β1 (Wang, Lu, et al. 2019).

#### 7.2.3 Genipin

Genipin is an aglycone derived from geniposide in *Genipa americana* and *Gardenia jasminoides* . It is a natural cross‐linker for proteins and has applications in drug delivery systems. Genipin also exhibits low acute toxicity (Yagupsky 2018). Genipin decreases the DNA affinity interaction of proteins p65 and p50. Thus, it aids in the suppression as well as degradation of other interconnected proteins, including IκBα, IKK‐α, and IKK‐β. It also phosphorylates IκBα (Wang et al. 2012). Genipin's anticancer potential is also limited to in vitro and in vivo studies (Luo et al. 2019; Cho 2022).

### 7.3 Terpenoids

Among naturally occurring phytoconstituents, terpenoids are the largest and most diversified group. They create the flavor, color, and scent of plants. The number of carbons created by the isoprene units they contain determines their classification. Terpenoids, a gaseous hydrocarbon with the chemical formula C_5_H_8_, are made up of these isoprene units (Cox‐Georgian et al. 2019). Terpenoids are further categorized into mono, sesqui, di, tri terpenoids, and carotenoid. Terpenoids are known to inhibit NF‐κB signaling in cancer therapeutics.

### 7.4 Sesquiterpenes

#### 7.4.1 Costunolide

Costunolide is a sesquiterpene lactone popular for its multiple therapeutic actions, encompassing inflammation‐modulating, antioxidative, antiallergic, and oncoprotective effects. It modulates intracellular signaling pathways and targets enzymes like NF‐κB (Kim and Choi 2019). Costunolide demonstrates NF‐κB modulating properties by phosphorylating the IκB protein (Koo et al. 2001). Costunolide regulates NF‐kB and TGF‐β_1_ signaling pathways to show inhibitory effects on pulmonary fibrosis (Liu et al. 2019).

#### 7.4.2 Ergolide

Ergolide is a fabulous phytochemical that could demonstrate strong effects even at lower concentrations, which makes it a good choice for a future drug candidate. Moreover, like other phytochemicals mentioned, it also possesses inflammation‐fighting capabilities, which it does by limiting the NF‐κB upregulation. This, in turn, suppresses the production of inducible nitric oxide synthase (iNos) and cyclo‐oxygenase‐2 (COX‐2). These limited factors are one of the primary contributors to limiting the inflammatory pathways (Whan Han et al. 2001). It also suppresses the working of NF‐κB assemblies and the deterioration of IκB (Whan Han et al. 2001).

#### 7.4.3 Helenalin A

This phytochemical is a toxic sesquiterpene‐lactone obtained from the *Arnica* plant species. Though it possesses toxicity, it is also a good candidate as it has shown anti‐inflammatory and antineoplastic effects in vitro studies. Helenalin inhibits NF‐κB and has potential medical applications, though it is not FDA‐approved (Perry et al. 2009). It does its job through the alkylation of the p65 domain, consequently obstructing the DNA interaction of the NF‐κB complex (Lyss et al. 1998). It could also be a good candidate for cancer treatment if further studied and successfully modified without compromising its medicinal effects.

#### 7.4.4 Humulene

Humulene, or α‐humulene, is a sesquiterpene found in hops and cannabis. It is known for its anti‐inflammatory and antimicrobial properties, though specific details about its mechanisms are less documented than other sesquiterpenes (Katsiotis et al. 1989). Humulene decreases the LPS‐triggered activation of the NF‐κB signaling pathway. Humulene is still merely a preclinical research molecule because there are no human clinical trials for cancer despite its intriguing anti‐cancer effects in lab and animal studies.

#### 7.4.5 Parthenolide

This is a bioactive sesquiterpene lactone found in feverfew (a common plant used for medicinal purposes). It is known for its anti‐inflammatory and anticancer properties, primarily through inhibiting NF‐κB potentiation. This phytochemical compound could inhibit the nucleocytoplasmic transport of p65, thus blocking DNA linking to the NF‐κB conjugate (Saadane et al. 2007). The compound parthenolide blocks NF‐κB and was studied to enhance cyclophosphamide's anti‐cancer effect on lung cancer cells in vitro and in vivo. The anti‐cancer effect included reduced proliferation and targeting the tumor microenvironment (Cai et al. 2024).

#### 7.4.6 Artemisinin (AT)

Artemisinin (AT) is another sesquiterpene lactone extracted from *Artemisia annua* . It is AT used as an antimalarial drug due to its potent activity against *Plasmodium* parasites. AT also exhibits anticancer properties. It shows anti‐cancer properties (Anwar, DasGupta, Shafie, et al. 2022) by inhibiting the LPS/cytokine‐driven activation of NF‐κB (Aldieri et al. 2003). AT has been tested in early Phase I and II clinical trials; however, no strong clinical evidence regarding the effectiveness of the compound is known. Although AT and its derivatives show promising anti‐cancer potential, the safe concentration required is in nanomolar doses. The inhibition of cancer requires micromolar doses, which can exert toxic effects on the nervous system (Genovese et al. 2000; Li et al. 2005).

#### 7.4.7 Valerenic Acid

It is a bicyclic sesquiterpenoid phytochemical in valerian root ( *Valeriana officinalis* ). It acts as a sedative by modulating GABA receptors, potentially aiding sleep and anxiety relief. It also has roles in BDNF expression and gastrointestinal motility. Along with this, valerenic acid is also another strong limiting agent for the NF‐κB and cytokine activation (Jang et al. 2008).

#### 7.4.8 Zerumbone

This phytochemical is a monocyclic sesquiterpene discovered in the rhizomes of *Zingiber zerumbet* . It exhibits anti‐inflammatory, antinociceptive, and antioxidant properties, making it helpful in managing inflammatory disorders like allergies and asthma (Su et al. 2021). It induces the phosphorylation of IκB proteins (Takada et al. 2005).

### 7.5 Monoterpenes

#### 7.5.1 Limonene

Limonene is a monoterpene found in citrus fruits (a class of fruits including lemons and oranges). Like other phytochemical compounds, it also possesses inflammation‐limiting, antioxidant and anticancer functions. Limonene is also used in aromatherapy for its mood‐enhancing effects (Perry et al. 2009). It could block the phosphate group transfer reaction of IκBα and p65 (Chi et al. 2013) and then ultimately disrupt the signaling mechanisms of the NF‐κB signaling pathway. It also acts as a mediator by limiting the availability of NF‐kB p65 subunit, which, in normal conditions, is translocated to the nucleus and disease‐related pathologies are observed (Yang, Chen, et al. 2021). This affect is achieved by its mechanism of prevention of phosphorylation (Chauhan et al. 2022). It also acts as the degradation of inhibitor complexes limiter. In the absence of these two effects, one could expect the transcription of proinflammatory genes (Chauhan et al. 2022). Through molecular docking‐based in silico studies, the binding of NF‐kB p65 with PI3K proteins has already been shown. These interactions result in the interference of their activation (Yang, Chen, et al. 2021). The pathway suppression of Limonene is dedicated to its inhibitory properties against upstream regulators of various pathways, including the PI3K/Akt/IKK/MAPK pathways (Younis 2020; Yang, Chen, et al. 2021).

#### 7.5.2 α‐Pinene

α‐Pinene is a terpene found in coniferous trees and herbs like rosemary. This is a multi‐source phytochemical, which is widely recognized as an indoor pollution causing agent. It is categorized as a monoterpene having two cyclic structures. α‐Pinene has multiple applications such as chemicals used as fragrances and flavor providers. Though obtained through multiple sources, pine trees are its primary producers, as one could understand by reading this phytochemical's name (Waidyanatha et al. 2022). It has anti‐inflammatory and antimicrobial properties and acts as a GABA receptor modulator. α‐Pinene is also known for its role in memory enhancement due to its acetylcholinesterase inhibitory activity (Mahmoudvand et al. 2016). α‐Pinene inhibits the NF‐κB/p65 protein complex translocation (Zhou et al. 2004). It gets easily evaporated in air due to its highly volatile nature, as suggested in published articles (Rastogi et al. 2001). As it keeps the tumor gene transcription factors within the cytoplasm trapped, the downstream events are prevented. This results in the prevention of tumor survival, inflammation, and metastasis (Zhou et al. 2004).

### 7.6 Diterpenes

#### 7.6.1 Acanthoic Acid

Obtained from the plant *Acanthopanax koreanum*, this phytochemical (i.e., acanthoic acid) is a pimaradiene diterpene. It has a diverse array of pharmacological activities, primarily through targeting various signaling pathways (Dou et al. 2022). Acanthoic acids reduce the augmentation of LPS‐driven IκBα phosphate group transfer. Thus, it inhibits the docking onto DNA to the NF‐κB system (Chao et al. 2005). Acanthoic acid and other diterpenes block the phosphorylation and degradation of IkB‐α, which, in normal scenarios, is well known to secrete NF‐kB for the process of nuclear translocation. As a result of the stabilization of IkBα, NF‐kB remains separated, within the cytosol of the cell, resulting in the failure of the activation of target genes (Través et al. 2014). In addition to this, Acanthoic acid could also inhibit the activation of several kinases, which are usually involved in the phosphorylation of p65 protein. Thus, its interference in the NF‐kB pathway is a crucial and help in the prevention of cancer activity (Través et al. 2014).

#### 7.6.2 Oridonin

Oridonin is a diterpene isolated from *Isodon rubescens*, and the phytochemical is studied and shown to possess inflammation‐modulating and antineoplastic attributes. This Chinese medicine has remained a subject of investigation in cancer research because of its medicinal properties. It acts by inhibiting signaling cascades associated with inflammation and cell proliferation, including the NF‐κB signaling pathway. Oridonin impedes the association of DNA with NF‐κB conjugate (Ikezoe et al. 2005). In mice models, its administration has been demonstrated to provide relief from carrageenan‐driven pleurisy. The effect is achieved by the activation of KEAP‐1/Nrf2 pathways. Actions of these pathways in downstream stages cause the inhibition of TXNIP/NLRP3 and NF‐kB cellular pathways (Li et al. 2021). From the available resources and information, it could be concluded that Oridonin is a good anti‐cancer, however, as it is in an earlier stage of research, more evidence is required to call it a medicine. Efforts are ongoing by researchers to make it more effective by preparing its derivatives or nano‐formulations to make it more soluble and more tolerant (Arefnezhad et al. 2025).

### 7.7 Triterpenes

#### 7.7.1 Lupeol

Lupeol is a triterpene having five cyclic rings, present in diverse botanical sources, it exhibits notable anti‐phlogistic, redox‐modulating, and oncopreventive bioactivities. It is often used in traditional medicine for its therapeutic benefits. Lupeol inhibits the phosphorylation of IκB proteins (Huang et al. 2005). Its effects is not direct, but has been demonstrated to exhibit chemopreventive properties, as reported in the studies involving experimental models (Fatma et al. 2024). Instead of a single‐step reaction, it helps in the prevention of carcinogenesis through multiple steps (Fatma et al. 2024). It works by the inhibition of key oncogenic pathways including NF‐kB, which is involved in cell survival, growth, and epithelial‐mesenchymal transition (Harikrishnan et al. 2020). At increased concentrations, it can inhibit the growth of new blood vessels, having the potential to be transformed into cancerous ones, a process known as angiogenesis.

#### 7.7.2 Betulin

Betulin is a triterpene found in the bark of birch trees. It has anti‐inflammatory, antiviral, and anticancer properties, making it a compound of interest for pharmaceutical applications. Betulin inhibits the phosphorylation and degradation of IKK protein. It also inhibits the degradation and nuclear translocation of p64 (Alakurtti et al. 2006). Betulin phytochemical and its closely linked botulin acid, a derivative, primarily targets cancer through the activation of the NF‐kB cascade. This reaction in turn inhibits survival, inflammatory response, and prevents anti‐apoptotic signal in cells that have the potential to become cancerous (Rabi et al. 2008). In experimental investigations involving the use of experimental models, commonly seen effects upon its administration include reduced cancerous cell proliferation, increased apoptosis, and decreased chemoresistance, instead of killing the cell directly. Like other phytochemicals, it also mediates primarily by showing cellular response of the reduction of p65's nuclear translocation (Shen et al. 2019).

#### 7.7.3 Ginsenosides

Ginsenosides are triterpene saponins found in ginseng (a Chinese phytochemical popular for its energy‐boosting properties). They have other Pleiotropic pharmacological responses too, comprising inflammation‐modulating, redox‐balancing, and neuroprotective activities, contributing to ginseng's traditional use as an adaptogen. These naturally obtained chemical modulators could show their effect on the NF‐κB pathway through the suppression of IκBα protein catabolism and IKKα upregulation (Hofseth and Wargovich 2007). This preventive action IkBα degradation and manipulation of IKK action in a combined fashion obstructs nuclear movement of NF‐kB subunits. This downregulates the expression of pro‐inflammatory cytokines like TNF‐α, IL‐6, and IL‐1β. Apart from this, it also lowers the COX‐2 and iNOS in cells, including both immune and non‐immune system components. Because of these mechanisms, ginsenosides aid in dampening increased NF‐kB activation in response to cellular stresses like oxidative stress. By limiting NF‐κB‐mediated survival signals, multiple ginsenosides increase the sensitivity of cancer cells to apoptosis and chemotherapeutic agents (Kannan et al. 2025).

#### 7.7.4 Glycyrrhizin

Glycyrrhizin is a triterpenoid saponin found in licorice root. It has anti‐inflammatory and hepatoprotective properties, though its use is limited due to potential side effects like hypertension. Glycyrrhizin could prevent the association of NF‐κB with DNA, thus modulating its activity. It could thus avoid induction of NF‐κB signaling through the phosphate group transfer or catabolism (or both) of IκB (Cherng et al. 2006). It can halt the binding of NF‐kB to nucleic acid (DNA). This action directly interferes with the transcriptional activation of pro‐inflammatory genes, along with those linked to cell survival. Those effects may arise from structural or redox‐sensitive changes of the NF‐kB's DNA binding domain. This mechanism has been observed with other similar inhibitors that target cysteine‐rich regions in the Rel homology domain of p65 (García‐Piñeres et al. 2001). Glycyrrhizin can also inhibit the induction of NF‐kB signaling by elevating the phosphate‐group transfer onto IkBα and its proteasomal degradation. These actions limit the release of NF‐kB dimers, and then their nuclear translocation happens (Gendy et al. 2023).

### 7.8 Carotenoids

#### 7.8.1 Lutein

Lutein is a xanthophyll carotenoid found in leafy greens. It is known for its antioxidant properties and is important for eye health, particularly in reducing the risk of macular degeneration. Lutein inhibits the translocation of p65 domains into the nucleus while triggering the catabolism of the IκBα protein (Izumi‐Nagai et al. 2007). From the reported experimental studies, it is predicted that Lutein may stabilize the catabolism of IκBα protein by limiting its phosphorylation through the modulation of IKK activity. The other proposed manner is that it modulates upstream kinase signals, which help in the maintenance of cytoplasmic sequestration of NF‐κB, thus preventing its over‐activation. Because of having a dual role, including NF‐κB translocation, and improving the degradation of IκBα, it restrains the NF‐κB pathway. This results in the contribution of anti‐inflammatory and cell protective effects in the retina and other tissues exposed to light‐induced and metabolic oxidative stress (Wu et al. 2024).

#### 7.8.2 Lycopene

Lycopene is a carotenoid pigment found in tomatoes. It has antioxidant properties which are correlated to a lowered chance of certain cancers and cardiovascular diseases. It modifies the NF‐κB signaling through the inhibition of phosphorylation, a type of post‐translational modification of IκB in cellular units and also by NF‐κB‐mediated gene expression (Assar et al. 2016). Through the inhibition of IKK‐linked IkBα phosphorylation, lycopene aids in the maintenance of NF‐kB in an inactive, cytoplasmic complex. Thus, it limits the nuclear translocation and DNA binding activity. It also reduces the expression of certain genes in which NF‐kB is seen as a mediator, thus it reduces the transcription of pro‐inflammatory cytokines, chemokines, adhesion molecules and certain enzymes in vascular endothelial cells, macrophages, and transformed epithelial cells (Rosenbaum et al. 2024). Lycopene has been tested in clinical trials and has shown protective effects in prostate cancer, but it has not been proved reliable anti‐cancer therapy (Kucuk et al. 2001).

#### 7.8.3 β‐Carotene

β‐Carotene is a carotenoid precursor to vitamin A, found in orange and other naturally obtained farming products. It acts as an antioxidant and is important for vision health and immune function. β‐Carotene facilitates the process of degradation of IκBs, thereby preventing nuclear movement of the p65 subunit and hindering the DNA‐binding ability of NF‐κB compound (Kalariya et al. 2008). It facilitates the process of degradation of those proteins belonging to the IkB family, the most common example is IkBα, thus demonstrating a modulatory effect on the NF‐kB signaling pathway. By supporting the timely turnover of IkBs, β‐carotene aids in the prevention of nuclear movement of p65 subunit. This reaction hinders the DNA‐binding ability of NF‐kB heterodimer and reduces the expression of downstream target genes involved in inflammation, cell survival and oxidative stress (Jung et al. 2008).

### 7.9 Non‐Dietary Polyphenols

#### 7.9.1 Obovatol

Obovatol is a lignan‐type polyphenol primarily isolated from the bark of *Ostrya* species, such as *Ostrya japonica* and *Ostrya virginiana* (Zuhrotun et al. 2023). It exhibits antioxidant, anti‐inflammatory, and antimicrobial properties, contributing to its traditional use in managing infections and inflammatory conditions (Balkrishna et al. 2025). Obovatol suppresses the translocation of NF‐κB to the cell's core. Along with it, it also inhibits the release of IκB, because of which the process of DNA binding gets inhibited and the activity of the NF‐κB pathway gets affected (Choi et al. 2007). Despite promising preclinical evidence demonstrating NF‐κB inhibition and anticancer activity, obovatol has not moved to clinical evaluations.

#### 7.9.2 Macranthoin G

Macranthoin G is a naturally occurring phenolic glycoside‐type phytochemical bioactive compound obtained from *Eucommia ulmoides* . It originates from the methyl ester of caffeic acid and a related compound called chlorogenic acid. When checked in PC12 cells, exposure to macranthoin G reduced cellular injury by downregulating the NF‐κB signaling cascade while improving the phosphate group transfer reaction of IκB, p38, and ERK. It inhibits DNA binding to NF‐κB and activates the post‐translational phosphate group transfer of IκB to modulate the NF‐κB pathway (Hu et al. 2014).

## 8 Limitations, Challenges and Future Perspectives of Anti‐Cancer Phytochemical Therapy

The fight against cancer was initiated with the efforts of the National Cancer Act of 1971, and despite decades of hard work and a few successes, the complete eradication of cancer still needs a lot of effort. Cancer is challenging to treat due to its complexity and the off‐target effects of known treatments. The chances of a new cancer medication being authorized are still slim, even though the amount of money needed for research and development to launch a fresh FDA‐approved medication has been steadily rising. Repurposing outdated medications, which is quicker and less expensive than creating new ones, is an intriguing substitute strategy. With the extra advantage that the safety issues are previously known, repurposed medications may help alleviate the scarcity of novel medications. However, in combination therapy, their interactions with other novel medications should be investigated. The development of naturally occurring phytochemicals has evolved as an adjuvant therapy in cancer treatment. The polyphenols are diverse in their chemical structure and biological effects. However, there are major limitations in administering these compounds. The limitations of phytochemicals as anti‐cancer agents will be discussed in this section.

The emergence of phytochemicals as anticancer agents requires sufficient evidence from clinical trials before they are approved as potent drugs. Although the discussed compounds in this review possess significant anti‐cancer effects via inhibiting the NF‐κB pathway, they have limitations to overcome before further clinical applications. Research has suggested roles of inflammation in the development of various diseases, including cancer. The inflammatory microenvironment is essential for the development of cancer. The NF‐κB pathway has a role in the development of an inflammatory microenvironment and the progression of cancer (Chrysanthakopoulos and Vryzaki 2023). The review has discussed in depth the role of phytochemicals in inhibiting the NF‐κB pathway; however, the compounds show promising anti‐cancer effects in vitro and animal studies, but clinical efficiency is limited to some compounds with minimal effects. The reason for limited performance in the clinical setup is attributed to poor aqueous solubility, low penetration in targeted cells, off‐target effects, and absorption by normal cells and potential toxic effects with increased doses (Choudhari et al. 2020).

Although phytochemicals have various limitations, research is focusing on removing the challenges. As bioavailability is one of the major challenges of phytochemicals, which affects stability, absorption, and metabolism, nanotechnology and lipid‐based formulations such as liposomal delivery, microencapsulation of compounds, and nano‐emulsion have emerged to overcome the challenges. The techniques are known to enhance the solubility, stability, and remove the off‐target bindings, which eventually reduces the quantity of phytochemicals needed to show the effect (Singh et al. 2019; Jafri et al. 2020). Apart from the challenges of bioavailability, optimizing and characterizing anti‐cancer properties of phytochemicals is a tedious process. Further advancements in analytical techniques and the development of software in computational biology may facilitate the identification of new compounds.

## 9 Conclusion and Future Perspectives

The NF‐κB pathway mediates various events, such as angiogenesis, tumor growth, metastasis, and treatment resistance. Genetic alterations that control NF‐κB activation may increase NF‐κB activity and significantly impact cancer development. In the fight against cancer and its treatments, molecular targets that aim to reduce NF‐κB activity have drawn significant attention. Potential medications can be made from phytochemicals, which are valuable resources. Complex molecules with a wide range of actions or particular ones can be abundant in plants. Their capacity to regulate other pathways, including PI3K, AKT, MAPK, and p53, adds to the therapeutic potential and complexity of NF‐κB‐targeted medications. The function of polyphenols in controlling the NF‐κB signaling pathway has been covered in recent research. These factors have led the scientific community to focus on polyphenols, which are present in various plants, as a possible substitute strategy for treating or preventing cancer. According to this review, polyphenols may prevent cancer by directly or indirectly modulating the NF‐κB pathway.

## Author Contributions

**Syed Tasqeruddin:** visualization, writing – review and editing, software. **Anas Shamsi:** conceptualization, methodology, validation, investigation, project administration, formal analysis, supervision, writing – original draft, writing – review and editing. **Moyad Shahwan:** investigation, conceptualization, project administration, writing – original draft, data curation. **Khuzin Dinislam:** conceptualization, methodology, visualization, writing – original draft, data curation.

## Funding

The authors are grateful to Ajman University, UAE, for supporting the publication through 2025‐IRG‐DRG‐10. The authors extend their appreciation to the Deanship of Research and Graduate Studies at King Khalid University, KSA, for funding this work through Small Research Group under grant number RGP.1/73/46.

## Conflicts of Interest

The authors declare no conflicts of interest.

## Data Availability

Data sharing not applicable to this article as no datasets were generated or analyzed during the current study.
